# 

**Published:** 1887-02

**Authors:** 


					﻿Vol. VII
FERBUARY, 1887.
NO. 2.
THE
HOMEOPATHIC pHYjSlClAfl,
A MONTHLY JOURNAL OF MEDICAL SCIENCE.
"He is a freeman whom truth makes free, and all are slaves beside."
"Seek the Truth: come whence it may, cost what it will."
EDITED BY
Edmund j. lee, m. d.,
AND
WALTER M. JAMES, M. D.
PHILADELPHIA:
No. 1123 8JPRTJCFC STREET.
new toisij-sz.i
A. I* CHATTERTON & COMPANY, Publisher’s Agents.
LONDON:
ALFRED HEATH & CO., Sole Agents foe Great Britain,
„ 114 Ebury 8treet, Eaton Square.
Yearly Subscription, $2.50, invariably in advance. Single numbers, 25 cts.
Entered at the Philadelphia Post-Office as Seoond-Class Mall Matter.
				

